# Correction: Association between serum uric acid, hyperuricemia and low muscle mass in middle-aged and elderly adults: A national health and nutrition examination study

**DOI:** 10.1371/journal.pone.0322650

**Published:** 2025-04-10

**Authors:** Laixi Kong, Yaqin Li, Rong Zhu, Maoting Guo, Yuqing Wu, Yuxin Zhong, Zhe Li, Zhenzhen Xiong

There are errors in the author affiliations. The correct affiliations are as follows:

Laixi Kong^1^, Yaqin Li^2,3^, Rong Zhu^4,5^, Maoting Guo^1^, Yuqing Wu^1^, Yuxin Zhong^1^, Zhe Li^6,7^, Zhenzhen Xiong^1^

**1** School of Nursing, Chengdu Medical College, Chengdu, Sichuan, China, **2** School of Nursing, The Hong Kong Polytechnic University, Chengdu, Sichuan, China, **3** School of Nursing, West China Hospital, Sichuan University, Chengdu, Sichuan, China, **4** The 3rd Affiliated Hospital Of Chengdu Medical College, Chengdu, Sichuan, China, **5** Pidu District People’s Hospital, Chengdu, Sichuan, China, **6** Mental Health Center, West China Hospital, Sichuan University, Chengdu, Sichuan, China, **7** Sichuan Clinical Medical Research Center for Mental Disorders, Chengdu, Sichuan, China.
